# Reliability of accelerometric assessment of balance in children aged 6–12 years

**DOI:** 10.1186/s12887-020-02073-1

**Published:** 2020-04-14

**Authors:** J. García-Liñeira, J. L. García-Soidán, V. Romo-Pérez, R. Leirós-Rodríguez

**Affiliations:** 1 Faculty of Education and Sport Sciences, University of Vigo, Campus a Xunqueira, s/n, 36005 Pontevedra, Spain; 2 Faculty of Physical Therapy, University of Vigo, Campus a Xunqueira, s/n, 36005 Pontevedra, Spain

**Keywords:** Accelerometer, Biomechanical phenomena, Gait analysis, Kinetics, Postural balance

## Abstract

**Background:**

Development and evaluation of an accelerometers technique for collecting data for asses balance had reported difficulty due to equilibrium reactions and continuous bursts. The aim of this study is to determine the reliability and internal consistency of accelerometric measurements, related to static equilibrium and gait in children aged 6 to 12 years.

**Methods:**

This descriptive and cross-sectional study involved 70 healthy children (50% girls) with a mean age of 9 years old. At the height of the 4th lumbar vertebra and directly on the skin, an accelerometer was placed on each participant. All of them had to complete four trials three times: balancing on one leg with eyes closed and eyes open, dynamic balancing on one leg on a foam mat, and normal gait.

**Results:**

Results show that tests performed in older children had higher internal consistency than those performed in younger children (vertical axis r = 0.82, sagittal axis r = 0.77, and perpendicular axis r = 0.74). Tests performed in children aged 8 years or older presented a strong correlation between trials (r > 0.71). The three static equilibrium tests obtained reliability values between 0.76 y 0.84. On the contrary, gait test obtained inferior and poorer results (0.6 < r < 0.71).

**Conclusions:**

This method of assessment obtained positive results as an instrument for the quantitative assessment of balance in school-aged children. Values obtained for the three one-leg balance and static tests,were more strongly correlated than the normal gait test for all axes.

## Background

The area of physical education pursues the development of fundamental psychomotor skills and abilities. In early stages of life, the balance has its own development with a strong reorganization at 6 years old [1]. This capacity depends on the senses (such as vision and proprioception), on vestibular system and the motor control system [2];which is necessary for the future cognition improvement, social interaction [3] and also other motor skills of great complexity for that range of age [4]. The experimental data demonstrate that, the first reference frame used for the organization of balance control during locomotion is the pelvis, especially in young children. Head stabilization during posturokinetic activities, particularly locomotion, constitutes a complex motor skill that requires a long time to develop during childhood. Throghout the study of the emergency of postural strategies, it is essential to distinguish between results that can be explained strictly by biomechanical reasons and those reflecting the maturation of the central nervous system [5].

Balance is one of the less studied and quantified skills in the school environment. In addition, balance assessments are usually based on qualitative methods, which are inefficient and have low reliability [6]. Reliable tests have been developed in a limited way, but they require to use an expensive force or pressure platforms, magnetic tracking, infrared emitter, electronic pressure sensitive walkway, or surface electromyographic recordings to determine the individual’s center of pressure (CP). This is a very loyable and valuable clinical indicator to identify relatively premature sensory-motor deficits [7, 8].

On the contrary, numerous studies have been performed to assess equilibrium in reference to the global behaviour of the individual and not specifically their centre of mass (CM) [7]. Accelerometry allows the analysis of specific aspects related to this factor and it has been proposed as a new implementation method due to the inexpensiveness, reliability, portability and comfortability for the evaluator [9, 10]. Numerous studies with accelerometers have evaluated balance and have been focussed on adult or elderly populations, especially in cases of risk of falling [11–13]. Balance accelerometric evaluation has been compared repeatedly with clinical trials and tests results, having positive results in different populations such as: (a) elderly people with a history of falls or a cerebrovascular accident, (b) children with dyslexia, (c) patients with Huntington’s and Parkinson’s disease and (d) patients with progressive cerebellar ataxia, and with vestibular disorders [14].

In reference to the child population, several authors have reported difficulty in the use of accelerometers for collecting data in short periods of activity (indispensable for the assessment of individuals at a young age), because of children equilibrium reactions are, unique and characterised as vigorous and producing “bursts” [15, 16].

Taking this into account, the current study was carried out with the aim to determine the reliability and internal consistency of accelerometric measurements of static equilibrium and gait in children aged between 6 and 12 years.

## Methods

### Study design and sample

This descriptive and cross-sectional study was performed using a convenience sample of healthy children. Participants who met any of the following exclusion criteria were unable to participate: (a) children with some developmental disorder; (b) children who were unable to walk independently or without external orthotics; (c) those who could not stand for 60 s or more; or, (d) children with any specific contraindications to the evaluation tests.

This study involved 70 healthy children (50% girls) with a median age of 9 years old (SD = 1.8). The objective of the selection process was to include five girls and five boys aged between 6 and 12 years old; which corresponds to the compulsory elementary school child period in Spain (before entering secondary education). Measures of weight and height were taken and body mass index (BMI) for each participant was calculated. For the balance measurements, an accelerometer was placed in the medial lumbar zone, specifically coincident with the fourth lumbar vertebra. According to the latest biomechanical findings, the lumbar vertebra has been demonstrated to reflect the behaviour of the CM [17].

In order to carry out measurements of the accelerations of the CM, each participant completed four trials, each of which were repeated three times (rest between measurements was no longer than the time required to prepare for the next trial). The following trials were performed: (a) balancing on one leg with eyes closed (OLCE); (b) balancing on one leg with eyes open (OLOE); (c) dynamic balancing on one leg on a foam mat, with eyes open to induce the onset of dynamic equilibrium reactions (DOL); and (d) normal gait (NG) to a cone located 10 m away (each participant must walk around the cone and return to the starting point).

The OLCE, OLOE and DOL trials had a fixed duration of 30 s. The duration of the NG trial varied depending on the time required by the participant to finish the circuit.

Participants were told that, if they suffered an imbalance in a monopodal stance that required them to use their other leg to support them, they should recover the requested position in the shortest time possible. All participants were instructed to choose the leg on which they make the support. For that, they were allowed to make previous attempts to make the selection (which they had to respect for all the tests).

All participants submitted the written informed parental consent prior to the start of the procedure and the ethical approval was obtained from the Commission of Ethics of the Faculty of Sciences of Education and Sport of the University of Vigo (Spain; number 3–0406-14).

### Procedure

The first step of the procedure was to explain the purpose of the study to the participants and their parents, and give them a brief description of what they were supposed to do. The parents of all participants signed an informed consent form in accordance with the Declaration of Helsinki (revised 2013).

All procedures performed in studies involving human participants were in accordance with the ethical standards of the Commission of Ethics of the Faculty of Sciences of Education and Sport of the University of Vigo (Spain; number 3–0406-14).

Once the informed consent form was signed, the participants’ data (full name and age) were collected. After that, the anthropometric measurements (weight and height) were obtained using a scale (SECA®, Berlin, Germany) and a stadiometer (SECA®). For both anthropometric measurements, the students were asked to remove footwear and any unnecessary clothing and stay barefoot.

By last, the accelerometer was placed on the participant’s body. The device was attached with adhesive tape to avoid displacement. The trial was explained to the participants, and they were accompanied to the corresponding measurement room to start the test.

The sequence of the trials was determined taking into account possible fatigue of the lower limbs. The trial order was OLCE-NG-OLOE-DOL-OLCE-DOL-NG-OLOE-DOL-OLOE-OLCE-NG, with each trial (OLCE, OLOE, DOL and NG) performed three times.

### Instrument and processing of data

The accelerometer GT3+ (Actigraph®, USA) was used. These accelerometers were chosen for being triaxial, and also because they were able to calculate the root mean square (RMS; three axis module vector) measured in units of gravity (G). Each accelerometer was initialised for data collection with the specific software. The data were processed by the software after each round of data collection.

From the gravity acceleration vector obtained by each accelerometer, the angles which mark the orientation of the participant are determined, where *A*_*x*_, *A*_*y*_, *A*_*z*_ are the accelerations for each axis and $\sqrt{x^2+{y}^2+{z}^2}$ is their module of the acceleration vector or RMS of accelerations (1), (2) and (3).

1 $$ axis\ 1: alpha\ \left(\alpha \right)=\arctan \left(\frac{A_x}{\sqrt{{A_y}^2+{A_z}^2}}\right) $$

2 $$ axis\ 2: beta\ \left(\beta \right)=\arctan \left(\frac{A_y}{\sqrt{{A_x}^2+{A_z}^2}}\right) $$

3 $$ axis\ 3: gamma\ \left(\gamma \right)=\arctan \left(\frac{\sqrt{{A_x}^2+{A_z}^2}}{A_z}\right) $$

Accelerometers provide data on body movements in three axes: (a) axis 1 corresponds to the acceleration in the vertical axis (transverse plane); (b) axis 2 is acceleration in the sagittal axis (coronal/frontal plane); and (c) axis 3 measured acceleration in the perpendicular axis (anteroposterior plane). The accelerometer measurements were configured for a time frame of 1 s.

Once the data was uploaded to the ActiLife software, the accelerometric signal was processed with a 50 Hz threshold filter. This threshold is effective in removing signal noise prior to statistical analysis. The signal noise can originate from the accelerometer if it is not fixed correctly to the participant (aspect that is solved with two actions: first, calibration of the device before each use and its correct fixation to the subject’s skin with hypoallergenic tape). Selecting a high or low sample rate can also alter the accelerometric record. This frequency must be 50 Hz for the study of postural control [18–20]..

### Statistical analysis

The internal consistency and reliability of the accelerometric measurement was evaluated using an average inter-item correlation test.

The first step was to check whether the signals detected by the inertial sensors were consistent between trials, both within and between subjects. The signals recorded by each sensor for all trials with the same subject were compared. The inter-item correlation coefficient was calculated for each sensor and for all signals recorded for each subject. This correlation served as an indicator of the degree to which the subject repeated the same accelerations between trials. The repeatability of the data for each individual was also evaluated.

All calculations were performed using SPSS for Windows version 17.0. Descriptive statistics were used as a measure of central tendency, including the standard deviation as a measure of dispersion and the 95% confidence interval. The Pearson’s r value was calculated to assess the correlation between the duration calculated using different sensors. The significance level was set at *P* < 0.05.

## Results

### Analysis of test-retest reliability and similarity of measurements

The average inter-item correlation test was used to determine whether the accelerations measured by the inertial sensors had internal consistency, i.e., if the waveform was consistent between trials for the same subject.

The results show that the tests performed in older children had higher internal consistency than those performed in younger children (vertical axis r = 0.82, sagittal axis r = 0.77, and perpendicular axis r = 0.74; Table 1). Tests performed in children aged 8 years or older presented a strong correlation between trials (r > 0.71 for all axes). In contrast, tests performed on children aged between 6 and 7 years showed a moderate correlation (0.56 < r < 0.7 for all axes). In relation to the different tests performed, the three monopodal equilibrium tests obtained higher correlation values (0.56 < r < 0.82 for all axes) that the proof of NG test (0.51 < r < 0.78 for all axes).

**Table 1:** Results of the test-retest reliability analysis for each axis and evaluation test

Test	Age group	Vertical axis	Sagittal axis	Perpendicular axis	Root Mean Square
OLCE	6 years old	0.61*	0.58**	0.53*	0.67**
	7 years old	0.59*	0.6**	0.51**	0.58*
	8 years old	0.61**	0.68**	0.64**	0.62**
	9 years old	0.81**	0.75***	0.79***	0.8***
	10 years old	0.79***	0.81***	0.95***	0.83***
	11 years old	0.73**	0.89***	0.82***	0.96***
	12 years old	0.74***	0.82***	0.9***	0.94***
OLOE	6 years old	0.59*	0.6**	0.61*	0.65**
	7 years old	0.64*	0.75***	0.73***	0.8***
	8 years old	0.68***	0.72***	0.71***	0.72***
	9 years old	0.73***	0.77***	0.81***	0.8***
	10 years old	0.7***	0.84***	0.72***	0.83***
	11 years old	0.82***	0.96***	0.72***	0.9***
	12 years old	0.83***	0.75***	0.94***	0.93***
DOL	6 years old	0.6**	0.76***	0.7***	0.74***
	7 years old	0.78***	0.8***	0.62**	0.75***
	8 years old	0.7**	0.85***	0.81***	0.86***
	9 years old	0.76***	0.63*	0.84***	0.86***
	10 years old	0.86***	0.93***	0.86***	0.9***
	11 years old	0.82***	0.87***	0.88***	0.9***
	12 years old	0.9***	0.96***	0.84***	0.89***
NG	6 years old	0.67**	0.65**	0.58**	0.6*
	7 years old	0.68**	0.59*	0.62*	0.67*
	8 years old	0.76***	0.59*	0.63**	0.67**
	9 years old	0.7***	0.78***	0.62*	0.7***
	10 years old	0.83***	0.76***	0.86***	0.81***
	11 years old	0.88***	0.8***	0.71***	0.86***
	12 years old	0.86***	0.8***	0.76***	0.78***

*OLCE* One leg with eyes closed, *OLOE* One leg with eyes open, *DOL* Dynamic equilibrium in one leg, *NG* Normal gait

*p < 0.05

**p < 0.01

***p < 0.001

It is also interesting to calculate signals similarities between subjects. The test calculates the correlation between each pair of signals and then calculates the average of the resulting correlations. These results indicate that accelerations in the sagittal (r = 0.92) and vertical (r = 0.85) axes, as well as their RMS (r = 0.81), showed the smallest variation between subjects (Table 2). In contrast, the values of acceleration in the perpendicular axis showed a correlation between subjects ranging from 0.62 and 0.73.

**Table 2:** Similarity of measurements between subjects

	OLCE	OLOE	DOL	NG
Vertical axis	0.73**	0.85***	0.89***	0.83***
Sagittal axis	0.81***	0.7**	0.92***	0.85***
Perpendicular axis	0.65*	0.7*	0.72**	0.76***
Root Mean Square	0.9***	0.88***	0.81***	0.86***

*OLCE* One leg with eyes closed, *OLOE* One leg with eyes open, *DOL* Dynamic equilibrium in one leg, *NG* Normal gait

*p < 0.05

**p < 0.01

***p < 0.001

### Reliability analysis between parallel tests

The Table 3 shows the ranges and mean values for each tests in each axis and their RMS. The results of average correlations coefficients were calculated over all subjects for the three trials of each task. The results of this analysis showed that the three static equilibrium tests obtained reliability values between 0.76 and 0.84. On the other hand, NG test obtained lower results (0.6 < r < 0.71).

**Table 3:** Ranges and mean values of each axis in each test (mean ± standard deviation and [confidence interval])

Variable		OLCE		OLOE		DOL		NG	
Vertical axis	Maximum	55.6 ± 11	[50.3–61.1]	42.1 ± 6.9	[39.5–43.8]	63.2 ± 14.3	[60.8–67.3]	79.2 ± 15.1	[72.4–81.1]
	Minimum	0 ± 0	[0–0]	0 ± 0	[0–0]	0 ± 0	[0–0]	0 ± 0	[0–0]
	Mean	7.5 ± 2.6	[5.2–9.8]	3.7 ± 1.4	[2.3–5.2]	8.6 ± 1.4	[5.6–11.6]	37.8 ± 0.8	[34.6–40.9]
Sagittal axis	Maximum	52.8 ± 11.4	[50.7–57.3]	47.7 ± 8.6	[44.3–51.7]	71.5 ± 13.4	[68.4–73.7]	40.8 ± 8.4	[37.6–43.9]
	Minimum	1 ± 0.9	[0.2–1.7]	0.1 ± 0.1	[0–0.5]	0.1 ± 0.1	[0.1–0.7]	2 ± 0.6	[0.8–3.5]
	Mean	12.4 ± 4.4	[10.1–14.8]	6.7 ± 3.4	[4.8–8.5]	11.7 ± 2.6	[8.9–14.5]	20.3	[18.6–22.1]
Perpendicular axis	Maximum	52.5 ± 9	[48.7–55.5]	35 ± 6.8	[33.5–41.4]	67 ± 11	[61.7–70.3]	50.9 ± 8.5	[47.7–53.3]
	Minimum	3.7 ± 2.1	[1.1–5]	0.1 ± 0.1	[0–0.2]	0.3 ± 0.2	[0–0.8]	4 ± 3.2	[3–5.2]
	Mean	19.5 ± 8.1	[16.1–19.9]	5 ± 2.6	[3.6–6.4]	8.2 ± 2.5	[5.9–10.5]	29.6 ± 5.1	[27.9–31.4]
Root Mean Square	Maximum	92.3 ± 19.5	[91.2–94.7]	70.6 ± 14.4	[66.8–73.9]	109.2 ± 23.9	[92.8–115.7]	96.5 ± 16.6	[88.5–101.2]
	Minimum	2.2 ± 2.5	[0.8–3.7]	0.2 ± 0.1	[0–0.5]	0.4 ± 0.2	[0–0.8]	5.9 ± 3	[4.2–6.8]
	Mean	19.5 ± 9.3	[15.5–23.6]	10.9 ± 6.6	[7.9–13.9]	19.7 ± 8.7	[14.7–24.7]	56.5 ± 11.1	[53–60]

*OLCE* One leg with eyes closed, *OLOE* one leg with eyes open, *DOL* dynamic equilibrium in one leg, *NG* normal gait

### Comparison of the tests in each repetition and correlation analysis

**Fig. 1:**
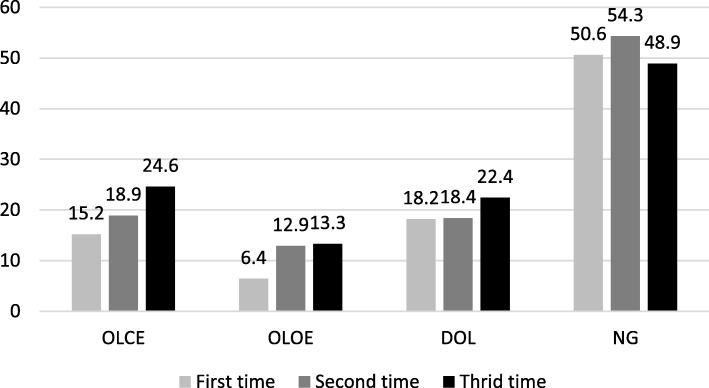
Evolution of Root Mean Square in each repetition of the tests. (OLCE: One leg with eyes closed; OLOE: one leg with eyes open; DOL: dynamic equilibrium in one leg; NG: normal gait)

The average acceleration results show that the RMS increased following the order of the trials (Fig. 1). In the OLCE, OLOE and DOL tests, accelerations in the sagittal and vertical axes gradually increased with each repetition.

The age of the participants was found to be correlated with the results of the three balance tests and the NG analysis regarding the accelerations in the three axes and the RMS (− 0.5 < r < − 0.8, *P* > 0.01). The subjects’ height was also found to be correlated with the results of the previous three tests and the NG analysis regarding to accelerations in the three axes and the RMS (− 0.5 < r < − 0.6, *P* > 0.001). Body weight and BMI were not correlated with acceleration.

Finally, despite the small sample size, no differences between males and females were detected in the differentiated gender analysis.

## Discussion

The objective of this study was to define the reliability and internal consistency of the accelerometric measurements of static equilibrium and gait in children aged between 6 and 12 years old. In order to the data, this method of assessment obtained positive results as an instrument for the quantitative assessment of balance in school-aged children. In the existing literature, we found no previous studies that performed accelerometric assessment in the lumbopelvic region to identify any difference between: (a) values recorded using different evaluation tests and (b) their different measurement reliabilities. Using a single accelerometer is common in studies of adult populations, however this study represents the first time that the reliability of a single device for assessing balance in children has been experimentally verified.

The results for the internal consistency and reliability of the instrument obtained in this study are positive. Nevertheless, it should be noticed that the results for children aged 6 and 7 years were only moderately positive, especially in the walking test. The normative description of the development of equilibrium and gait patterns throughout childhood maturation is complex; and it is closely related to the age of the individual, especially during the first years of independent walking [21].

By school age, children have already strengthened their gait and their ability to maintain static equilibrium. Thus, accelerometry could be used to study large groups of children, therefore future studies should establish the normative values of acceleration in: (a) static, (b) dynamic equilibrium and (c) walking.

Previous studies have provided reference databases for gait in children including: (a) temporal distance, kinematic and dynamic gait parameters of 10 toddlers aged 13.5 to 18.5 months old [22]; (b) ground reaction force patterns of more than 7000 children aged 1 to 13 years old [23]; and (c) the kinematic and dynamic parameters of 20 Chinese children aged 7 to 12 years old [24]. All of these three studies were based on the study of pressure centre with force platforms. These three studies were based on the study of the pressure center with force platforms. The pressure center is an indirect measure of the equilibrium reactions of the human body. However, the displacement of the center of gravity (CG) is a direct measure of biomechanical reactions against gravity [25]. This paradigm shift occurred after the definition of the multisegmental concept of equilibrium that defines the body as a system of rigid bodies, whose CG is the average of all the centers of mass of said segments [26].

Therefore, for a person to have a healthy control of balance (that is, to avoid falls) the determining aspect is keeping the CG under control. Such CG control can be automatic (involuntary) during activities of daily living, including activities such as walking, climbing and descending stairs, bending over or performing transfers sitting and standing, and vice versa; or voluntary, in the face of disturbances such as tripping and slipping [27].

In the field of research, the balance is usually evaluated by using force platforms. These instruments record the displacement of the pressure center, which, as mentioned, is an independent parameter of the CG and of the overall behavior of the body in the three planes of space. This parameter is subject to the inverted equilibrium pendulum theory [28], which is valid for movements that do not imply changes in the support area, it is inadequate for a holistic evaluation of the postural control system and all the strategies that this system uses to maintain balance [29, 30].

Alternatively, kinematic instruments, such as accelerometers, allow equilibrium to be objectively studied through GC analysis without great financial expenses on measurement devices, or complex data analysis processes [31, 32].

Acceleration results were increasing for all equilibrium tests as the three attempts of each test were performed. A plausible explanation for these accelerometric values is the appearance of fatigue [33], which occurs mainly in the stabilising muscles of the lower extremities (especially the hip abductors and stabilising ankle muscles), which alters the base of support and forces a readjustment of the trunk stabilising muscles (the abdominal muscles and the paravertebral musculature).

Despite the aforementioned, this phenomenon was not observed in the gait test. While NG fatigue does not appear as quick as it appears during equilibrium tests, notwithstanding it is a more complex activity than the static monopodal. In addition, it is a dynamic activity in which biomechanical actions are sequenced and coordinated between different muscle groups.

In relation to the accelerometric analysis of gait, the values for the RMS and the accelerations produced in the sagittal plane stand out as being particularly important. Both of them are in agreement with the existing literature.

Accelerations in the mid-lateral axis and the magnitude of the RMS of the accelerations have been strongly associated with the risk of falling in adults [14, 34]. This is relevant because it has been determined that falls are the most common injury mechanism in all age groups during childhood; and the origin of these falls: (a) the lack of sleep, (b) lack of concentration and (c) the deficit in the development of motor skills [35].

Studying the acceleration module is a constant in studies based on accelerometery, and measuring the magnitude of the movement has been used in almost all studies based on accelerometric analysis since this method was first introduced as a tool for assessing balance, both static and dynamic [10, 20, 36–38].

We should point out that the sample size is not sufficient to generalise the results obtained in the current study to the child population; however, it does confirm the reliability and consistency of static balance assessment instrument to carry out future studies that include normative values of acceleration and their evaluation percentiles according to age.

An important limitation of this study is that the results obtained do not allow us to describe how postural control systems work to maintain balance from a physiological point of view. Accelerometry is an indirect measure of the efficiency with which the central nervous system integrates information from the environment and from the subject themselves in order to maintain balance.

In the future, the possibility of expanding the sample to more specialised populations should be explored, including patients with neurological diseases such as cerebral palsy and muscular dystrophy.

The possibility of designing specific tests with accelerometric variables that would display the great deterioration in these populations should be considered. This would bring us the identification of patients in the early stages of these pathologies, as well as quantitatively evaluate specific interventions for early treatment.

It would also be of great interest to carry out a longitudinal study that relates variations in body composition with gait stability, and how these variables change as psychomotor maturation progresses. Such research would allow us to determine and compare the parallel evolution of body fat and muscle percentages with the kinematic parameters of balance and gait. In addition, the potential compenses of children for maintaining the balance should also be studied: with the use of a second Actigraph placed, for example, on the ankle or on upper limb.

## Conclusions

In view of the data obtained, we assert this method of assessment obtained positive results as an instrument for the quantitative assessment of balance in school-aged children.

The results show that tests performed in older children for the vertical, sagittal and perpendicular axes have greater internal consistency than those performed in younger children. The tests performed in children aged 8 years or older showed a strong correlation for all axes between trials.

The values obtained for the three one-leg balance and static tests were more strongly correlated than those obtained for the normal gait test for all axes.

## Data Availability

The database used to carry out this work is in the possession of the authors and will be provided to whoever requests it.

## Abbreviations

CM: Centre of mass
SD: Standard deviation
BMI: Body mass index
OLCE: One leg with eyes closed
OLOE: one leg with eyes open
DOL: dynamic balancing on one leg
NG: Normal gait
RMS: Root Mean Square
G: units of gravity
A: acceleration
CG: Centre of gravity
